# Transmural Extent of Hyperenhancement in ST-Segment Elevation Myocardial Infarction: An Unreliable Measure of Acute Viability

**DOI:** 10.1016/j.jacadv.2023.100328

**Published:** 2023-05-24

**Authors:** Casper W.H. Beijnink, Jamal N. Khan, Alexander Hirsch, Jagat Narula, Laura Rodwell, Albert C. van Rossum, Niels van Royen, Gerry P. McCann, Robin Nijveldt

Primary percutaneous coronary intervention (PCI) is the first-choice treatment in patients presenting with ST-segment elevation myocardial infarction (STEMI) within 12 hours of symptom onset. Viability testing to guide revascularization decisions in patients presenting late after STEMI is often assessed using late gadolinium enhancement (LGE) cardiovascular magnetic resonance (CMR) imaging. Myocardial segments with >50% transmural extent of hyperenhancement (TEH) are generally considered nonviable and unlikely to benefit from revascularization, in chronic coronary syndromes.^1^ In patients who were scanned in the (sub)acute phase, however, this paradigm has been challenged in a small study in revascularized STEMI patients.^2^ We aimed to examine the TEH on CMR in the acute phase and at 4 months after revascularization in 2 large multicenter cohorts of patients with STEMI undergoing primary PCI, including a small subset of patients who did not undergo revascularization.

In our analysis, 183 patients were selected from prospective randomized clinical trials, namely PREDICT MVO (Pressure-Flow Measurements Directly after Primary PCI to Predict Late Occurrence of Microvascular Obstruction)^3^ and HEBE (Multicenter, randomized trial of intracoronary infusion of autologous mononuclear bone marrow cells or peripheral mononuclear blood cells after primary PCI),^4^ in which primary PCI was performed within 12 hours after symptom onset in patients with their first STEMI. Both studies were approved by the local ethics committee of the Amsterdam University Medical Center (Amsterdam, the Netherlands) and conducted in accordance with the Declaration of Helsinki. Patients were included if both the baseline and follow-up CMR scan data were available. Baseline CMR (during index STEMI episode) was performed at a median of 4 days post-STEMI (IQR: 3-5 days) and follow-up CMR was performed at 124 days post-STEMI (interquartile range 116-129 days). In total, 16 segments could not be analyzed due to artefacts, in a total of 3 patients. Three patients were not reperfused. All 3 underwent baseline CMR at 4 days post-STEMI. Follow-up CMR was performed at 4 months in 2 patients and 4 years in 1 patient.

The CMR protocol included long-axis and short-axis steady-state free-precession cine and LGE imaging in identical orientations, segmented according to the American Heart Association 16 segment left ventricular (LV) model. Infarct size on LGE imaging was semiquantitatively determined with an identical approach for both CMR scans of each patient, using the full-width at half maximum technique^3^ or 5 standard deviations cutoff over healthy myocardium.^4^ Each myocardial segment received a grade expressing the TEH, where grade 0 = 0%, grade I = 1% to 25%, grade II = 26% to 50%, grade III = 51% to 75%, and grade IV = ≥75% TEH. Transmural infarction was defined as 51 to 100% TEH, that is, grades III-IV. Results were analyzed using SPSS (IBM SPSS Statistics, Version 25).

Categorical values were compared using chi-squared and McNemar tests for related samples. To assess if crossover to a lower transmurality category had occurred within each baseline class, a binomial test on a single proportion was performed, comparing the proportion of patients who moved at least one class lower against the null hypothesis of no cross-over.

The mean age of patients with STEMI was 56.3 ± 0.7 years. At baseline, LV ejection fraction was 45% ± 9%, acute anterior infarction was present in 101 (55%) patients who were reperfused and 2 (67%) patients who were not reperfused. The mean infarct size of reperfused cases was 18.1% ± 10.1% of LV and 18.6% ± 14.7% of LV for non-reperfused cases at baseline and, respectively, 13.5% ± 7.7% of LV and 9.7% ± 6.7% of LV at follow-up.

**Figure 1:**
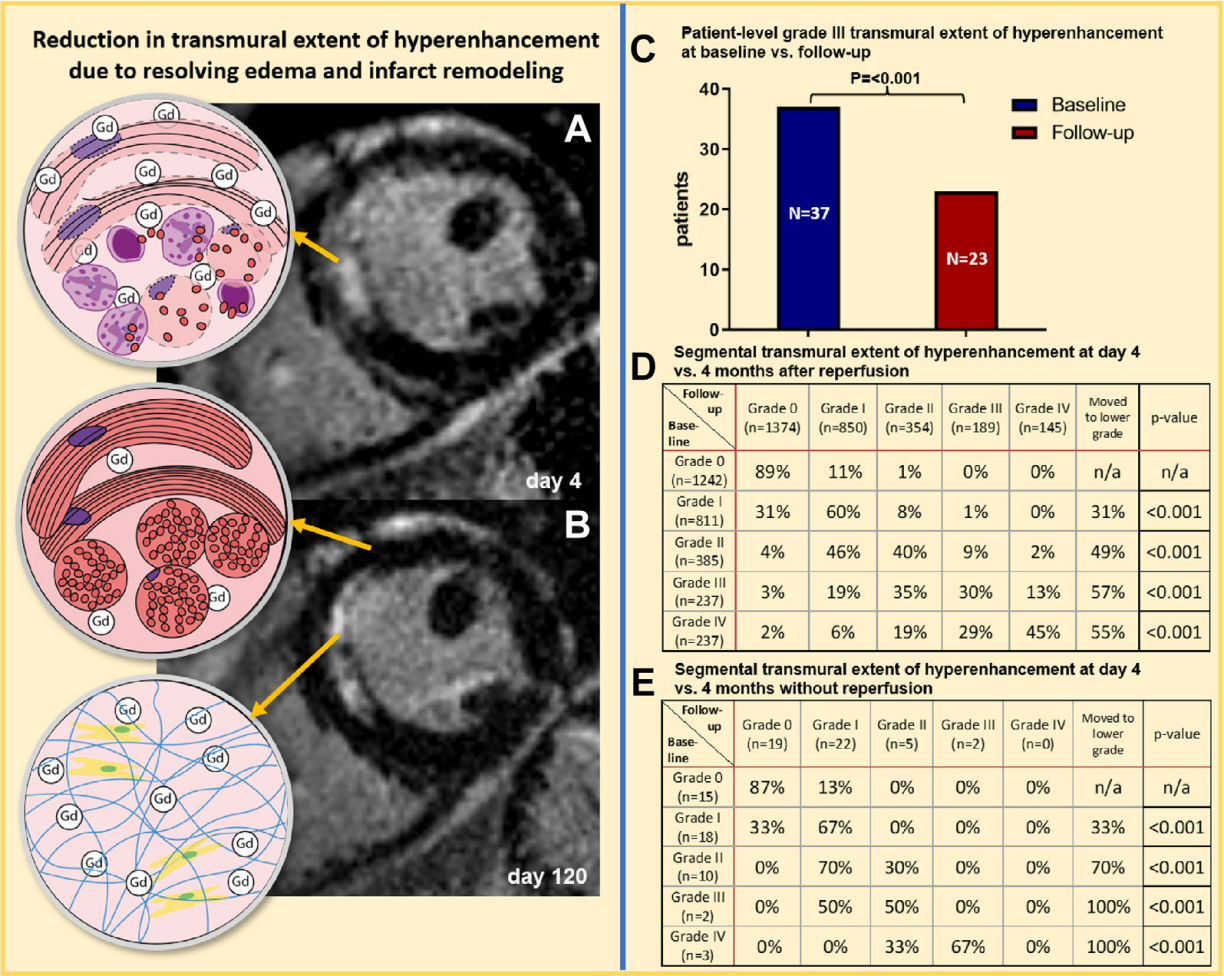
Transmural Extent of Hyperenhancement at 4 Days and at 4 Months Follow-Up **(A)** Example of an anterior myocardial infarction at day 4 after reperfusion demonstrating near complete transmural late gadolinium enhancement (LGE) **(white area, arrows)** on CMR imaging. **(B)** Corresponding LGE CMR image in the same patient at follow-up demonstrating reduced transmural extent of hyperenhancement. **(C)** Decrease in the number of patients with grade III transmural extent of hyperenhancement on CMR at baseline and in follow-up. **(D** and **E)** Transmural extent of hyperenhancement on CMR at baseline compared to follow-up on reperfused **(D)** and nonreperfused **(E)** left ventricular myocardial segments. CMR = cardiovascular magnetic resonance; LGE = late gadolinium enhancement.

On a segmental basis, in the overall cohort and reperfused cohort, the number of segments with TEH >25% (ie, grades II-IV) decreased from baseline to follow-up CMR (Figure 1D). A significant reduction in TEH grade occurred for all grades. Of all segments with baseline grade III TEH, 57% of segments had a lower TEH grade at follow-up. The majority of segments (55%) in patients with baseline TEH grade IV had a lower TEH grade at follow-up. However, 53% of those segments were classified as TEH grade III, remaining transmural. A reduction in TEH grade was seen in the smaller, nonreperfused cohort too, despite the lower number of included segments (Figure 1E).

Importantly, in patients (n = 37) with at least 1 segment with baseline grade III TEH (but not grade IV), 38% had exclusively grade 0-II TEH at follow-up (*P* < 0.001). This is in keeping with infarct resorption and functional improvement in segments which on their baseline scan would have been considered transmurally infarcted and nonviable (Figure 1C). These 37 patients would likely have been denied revascularization based on conventional LGE-based viability testing. This can be explained by the presence of myocardial edema in ischemic segments, which is known to cause an overestimation of the true necrosis extent on LGE acutely post infarction. The degree of edema, TEH, and infarct size thus resolves significantly over the first 4 months post infarct (Figures 1A and 1B) and functional recovery has been shown to occur in a substantial proportion of dysfunctional segments with acutely transmural hyperenhancement on LGE.

In conclusion, we demonstrated that LGE-assessed TEH in the acute phase post-STEMI decreases significantly by 4 months. This includes LV segments with 50% to 75% TEH, those traditionally considered nonviable and unlikely to benefit from revascularization. Our findings should discourage clinicians from using myocardial viability testing and a 50% TEH cutoff for guiding revascularization decisions in the acute phase after STEMI.
